# Clinical evidence-guided network pharmacology analysis reveals a critical contribution of β1-adrenoreceptor upregulation to bradycardia alleviation by Shenxian-Shengmai

**DOI:** 10.1186/s12906-019-2769-0

**Published:** 2019-12-10

**Authors:** Jiaming Gao, Taiyi Wang, Xi Yao, Weiwei Xie, Xianru Shi, Shuang He, Tao Zhao, Chunhua Wang, Yan Zhu

**Affiliations:** 1 State Key Laboratory of Component-based Chinese Medicine, Tianjin University of Traditional Chinese Medicine, Tianjin, 300193 China; 2 Research and Development Center of TCM, Tianjin International Joint Academy of Biotechnology & Medicine, Tianjin, 300457 China; 3 Xian Buchang Chinese Medicine Cardio Cerebral Disease Hospital, Xian, China

**Keywords:** Traditional Chinese medicine, Shenxian-Shengmai oral liquid, Bradycardia, β1-adrenergic signaling, Network pharmacology, Meta-analysis

## Abstract

**Background:**

Shenxian-Shengmai (SXSM) Oral Liquid is a CFDA-approved patent Chinese Herbal medicine, which has been clinically used for the treatment of bradycardia. However, its active components and action mechanism remain to be established. The present study aimed to evaluate the efficacy of SXSM on bradycardia and to identify the possible active components and their pharmacological targets for this action.

**Methods:**

A literature-based meta-analysis was performed to evaluate the clinical efficacy of SXSM on bradycardia, which was confirmed by a rat ex vivo cardiac model. Network pharmacology analysis was then conducted to reveal the potential targets of SXSM active components and their anti-arrhythmia mechanisms. Finally, the identified drug-target interaction was confirmed by immunofluorescence assay in cardiomyocyte.

**Results:**

Meta-analysis of the available clinical study data shows that Shenxian-Shengmai Oral Liquid has a favorable effect for bradycardia. In an ex vivo bradycardia model of rat heart, SXSM restored heart rate by affecting Heart rate variability (HRV) which is associated with autonomic nervous system activity. A drug-target-pathway network analysis connecting SXSM components with arrhythmia suggested that a prominent anti-arrhythmia mechanisms of SXSM was via β1-adrenergic signaling pathway, which was subsequently validated by immunofluorescence assay showing that SXSM indeed increased the expression of ADRB1 in cultured cardiomyocytes.

**Conclusion:**

By combining approaches of clinical evidence mining, experimental model confirmation, network pharmacology analyses and molecular mechanistic validation, we show that SXSM is an effective treatment for bradycardia and it involves multiple component interacting via multiple pathways, among which is the critical β1-adrenergic receptor upregulation. Our integrative approach could be applied to other multi-component traditional Chinese medicine investigation where ample clinical data are accumulated but advanced mechanistic studies are lacking.

## Background

Bradycardia, a multi-factorial concomitant health problem of cardiac vascular diseases (CVDs), is defined as heart beat lower than 60 bpm. It was divided into sick sinus syndrome (SSS), escape rhythm, atrioventricular block (AV block) and intraventricular block [1]. This irregularity of heart rhythm may be either pathological or physiological. Extensive exercise causes a lower heart rate of 30–60 bpm in overall 50–80% athletes [2]. Although congenital structural abnormalities always lead to conduction dysfunction, bradycardia may also occur in the normal heart as people growing up [3]. Accumulating evidence has indicated that arrhythmia is one of the common complications in many brain or heart-disease patients now. Accompany with coronary artery disease, cardiac hypertrophy, cardiac infarction, and stroke [1, 4, 5]. Currently, bradycardia treatment consists of implanting Cardiac implantable electronic device (CIED) and taking chemical drugs. Although pacemaker is clinically the most popular treatment, expensive operative cure is not suitable for all patients especially the old and the weak. In addition, device infection has become a major problem which results in another huge burden on the patients who paid an expensive implantation surgery costs already [6].

Recently, medicinal herbs have received considerable attention as treatment alternatives for arrhythmia [7]. Traditional Chinese Medicine (TCM) have been used for centuries. Compared with western drugs, herbal medicine is more accessible, less expensive, and mostly with fewer side effects. Clinical reports suggested that Shenxian-Shengmai (SXSM) oral liquid, a CFDA-approved patent Chinese medicine, was effective in treating bradycardia [8, 9]. SXSM formula consists of eight herbal components including Radix Ginseng Rubra (hongshen), Herba Epimedii Brevicornus (yinyanghuo), Fructus Psoraleae (buguzhi), Fructus Lycii (gouqizi), Herba Ephedrae Sinicae (mahuang), *Asarum heterotropoides* (xixin), Radix Salviae Miltiorrhizae (danshen), and Hirudo (shuizhi). Prior studies revealed that therapeutic effect of SXSM on chronic arrhythmia was related to the elevation of Na^+^-Mg^2+^/Ca^2+^-Mg^2+^-ATPase activity and increased expression of Cx43 and Kir2.1 protein [10], increased expression of acetylcholinesterase, reduced level of nicotinic receptor and improved ATP supply [11]. In addition, SXSM was recently reported to protect heart function in Ischemia/Reperfusion injury [12]. To understand the chemical basis of SXSM’s anti-arrhythmia activity, we previously identified a total of 64 compounds in SXSM by UPLC-QTOF-MS/MS and quantified 10 of the major constituents by UPLC-DAD [13]. However, due to the lack of disease-targeted active component identification, the pharmacological mechanisms of SXSM remain to be elucidated.

In this study, clinical evidence of SXSM was evaluated with a meta-analysis of all published reports up to date. Then, a chemical database of SXSM was constructed using data from a variety of TCM database resources and our validated experimental results. Network pharmacology analysis was preformed to identify potential drug-disease target relationship between SXSM components and bradycardia. The efficacy of SXSM in alleviating drug-induced arrhythmias was then confirmed in ex vivo cardiac model. The major network pharmacology-predicted mechanism of SXSM action was finally confirmed by immunofluorescent assay in cardiomyocytes.

## Methods

### Chemicals and reagents

Shenxian-Shengmai (SXSM) oral liquid was obtained from Shanxi Buchang Pharmaceutical Co., Ltd. (Shanxi, China, CFDA approval No. Z20080183 and lot No. 107582913146). Dulbecco’s modified Eagle’s medium (DMEM), fetal bovine serum (FBS), L-glutamine, penicillin, and streptomycin were purchased from Gibco (NY, USA). DMSO was purchased from Solarbio corporation (Beijing, China) and other reagents, including NaCl, KH_2_PO_4_ and MgSO_4_, were purchased from Sigma Chemicals (St. Louis, MO USA). Donkey anti-Rabbit IgG H&L (Alexa Fluor® 555) and rabbit anti-beta 1 Adrenergic Receptor antibody were purchased from Abcam corporation (Shanghai, China). Isoproterenol was purchased from Meilun biological corporation (Shandong, China).

### Animals

Adult Sprague-Dawley (SD) rats (males, 8 weeks old, weighting 200 g ± 30 g) were purchased from Beijing Vital River Laboratory Animal Technology Co.,Ltd. (Beijing, China, Certificate no.: SCXK Jing 2016–0006). The rodents were randomly divided into three different groups (control, positive control and SXSM) and housed in 480*300*160 mm cages at a temperature of 22 °C ± 2 °C, and a humidity of 40 ± 5%, under a 12 h light/dark cycle, and received standard diets and water ad libitum. All experiments were reviewed and approved by the Committee of Ethics on Animal Experiments at the TJAB (Permit Number: TCM-LAEC2014004) and were carried out under the Guidelines for Animal Experiments at the Tianjin University of Traditional Chinese Medicine. Rats were euthanatized by quick bleeding (within 10 s) through cutting off the coronary artery while the animals were in deep anesthesia (sodium pentobarbital) to reduce animal suffering.

### Meta-analysis of clinical studies

#### Database and search strategies

Following search terms were used as retrieval keywords: “Shenxian-Shengmai” OR “SXSM” OR “SSOL” OR “Shenxian-Shengmai oral liquid” OR “Shenxian-Shengmai oral solution” in combination with “bradycardia” OR “bradycardia” and “randomized controlled trial”. Following electronic databases (dated from January 2010 to expiration date as September 2018) were searched: China National Knowledge Infrastructure (CNKI), VIP Database, Wanfang Database, PubMed, Web of Science, SinoMed, and IPA Database. The search was limited to English and Chinese papers and, whenever possible, MESH terms were used. The reference lists of the included original papers were also examined. In addition, clinical trials were searched on the World Health Organization International Clinical Trials Registry Platform. For further analyzing clinical information, a reference bibliography of the included studies was summarized and can be searched in supplementary materials [14–16].

#### Inclusion and exclusion criteria

Based on the Cochrane Collaboration Handbook standards, the following criteria were formulated for the selected literatures [17]. The criteria conclude: duplicate publications, descriptive studies, animal research, reports without statistical indicators and reviews. Patients with bradycardia were eligible to be included. All the participants had to meet at least one of the current or past diagnostic criteria of bradycardia, such as Guidelines for diagnosis and treatment of common cardiovascular and cerebrovascular diseases in China of functional classification and electrocardiogram assessment. A meta-analysis was performed if the intervention, control, and outcomes were the same or similar. All the patients that studied prescriptions based on SXSM alone compared with other formula medicine or western medicine, namely amiodarone [18], were included. There were no restrictions on population characteristics or publication type. The primary outcome measure was the 24 h dynamic electrocardiogram after 4 weeks of treatment with SXSM in oral, and adverse drug reactions (ADR). The publication with more complete information was included in consideration of one identical clinical trial occurred in duplicated publications. In addition, for insurance we also searched the reference lists of all full text papers for additional relevant data.

#### Data extraction and risk of bias assessment

Data were extracted from the eligible papers screened on basis of assessing criteria by two reviewers, separately [19]. Extracted parameters included: a) the title of the study, b) first author’s name, c) year of publication, d) article source, e) sample size, f) groups, g) diagnosis standards, h) details of methodological information, i) dose form and treatment duration, j) the details of the control interventions, k) outcomes, l) adverse effects for each study. Disagreements were resolved by discussion. As for the methodological quality of the included trials was assessed according to the Cochrane Handbook for Systematic Review of Interventions, Version 5.1.0 [17]. The quality of all the included trials was categorized as three classes: low, unclear, or high risk of bias following seven criteria: random sequence generation (selection bias), allocation concealment (selection bias), blinding of participants and personnel (performance bias), blinding of outcome assessments (detection bias), incomplete outcome data (attrition bias), selective outcome reporting (reporting bias), and other sources of bias (More details in supplement materials).

#### Statistical analysis

RevMan 5.1.0 software, provided by the Cochrane Collaboration, was used for data analysis. Dichotomous data were presented as the odds ratio (OR), and continuous outcomes were presented as the weighted mean difference (WMD) or standardized mean difference (SMD), and 95% confidence intervals (95% CI) were calculated for both types of data. The statistical heterogeneity was presented as significant when the I squared (I^2^) value exceeded 50% or *P* < 0.1. In the absence of significant heterogeneity, the data using the fixed effects model (I^2^ < 50%) were pooled. If there was significant heterogeneity, the random effects model (I^2^ > 50%) [17] were used. Publication bias were evaluated using funnel plot analyses whether small sample effect was found. Heterogeneity was examined using the I-squared (I^2^) index. I^2^ values greater than 50% were considered indicative of high heterogeneity [20].

### Network pharmacology analysis

#### Database construction

The main source of disease targets was obtained from IPA database (http://www.ingenuity.com). Additional databases such as GeneCards, MalaCards, OMIM, and NCBI gene were manually searched [21, 22], portion of targets subtracted were added to complement the omissions. Duplicate genes were removed automatically. Information on TCM ingredients compound were retrieved mainly from literature and were also mined from several online TCM databases, including TCMSP, TCM Database@Taiwan, and TCMID [23–25]. Because of a compound has more than one chemical synonym, we discerned them by CID or CAS number on PubChem website (http://pubchem.ncbi.nlm.nih.gov/), which are recognized by IPA.

#### Network establishment and analysis

Three datasets, ①SXSM ingredients, ②arrhythmia-associated targets, ③SXSM’s major ingredients and their corresponding targets, were constructed. ClueGo module in Cytoscape software and “Build-Path Explorer” module in IPA were used to build connection and predict the interaction among compounds, targets and disease. Each compound or target were defined as a single node, and edge represents interaction. In this study, the algorithm of the network analysis was based on Fisher’s exact test with the enrichment score of *P*-values. “Overlay-Canonical Pathway” module was used to find the resulting canonical pathways. “Network analysis” module was utilized to analyze the correlation degree of the network which we established before. “Path designer” module was performed to beautify the network [26, 27].

### Isolated heart reperfusion and electrical signal recordings

Sprague Dawley (SD) rats were anesthetized by intraperitoneal injection of sodium pentobarbital (50 mg/kg). While aorta was clamped with a hemostat, hearts were quickly removed to pre-cooled K-H solution containing: 120 mM NaCl, 4.7 mM KCl, 25.0 mM NaHCO_3_, 1.2 mM KH_2_PO_4_, 1.2 mM MgSO_4_, 11.1 mM glucose, 2.0 mM Na-pyruvate and 1.8 mM CaCl_2_. The isolated heart was hung on Langendorff perfusion system and perfused with K-H solution equilibrated with 95% O_2_ and 5% CO_2_ mixed gas firstly and three electrodes were fixed on left ventricle, right atrium and the earth. When quickly reached a constant pressure at 70–80 mmHg, heart rate and electrocardiogram were measured. The bradycardia model was created by perfusing the heart with 100 nmol/L acetylcholine for 1 min, which caused a significant decrease in heart rhythm [28]. SXSM was administered at a high dose of 1 mg/ml. The raw ECG data were extracted from the telemetric recording using a custom software (BI9800 Biomedical Instruments, China) for Heart Rate Variability (HRV) measurements. The data were edited to manually remove instrumental and physiological artifacts. The following power spectral variables were determined: high-frequency (HF) component (0.15–0.4 Hz, a marker of the parasympathetic tone), low-frequency (LF) component (0.04–0.15 Hz, possibly correlated with sympathetic tone or to autonomic balance), and the ratio between LF and HF powers (LF/HF, index of the interaction between sympathetic and vagal activity).

### Immunofluorescence assay (IF)

Validation of the influence of SXSM on β1-adrenergic signaling pathway was performed by immunofluorescence assay with Human H9C2 cardiomyocytes as we previously described [29]. Cells were divided into 4 groups: control (blank), isoproterenol (positive drug) and SXSM high and low dose groups. H9C2 cardiomyocytes were seeded in 96-well plates at a density of 1.0 × 10^4^ cells/well and maintained at 37 °C with 5% CO_2_ for 24 h. DMEM with 10% FBS was replaced to serum-free medium when cells were grown to approximately 80% confluences. After drug treatment, cells were incubated at 4 °C overnight with anti-beta 1 Adrenergic Receptor antibody (1:100 dilution) for 24 h and then rinsed with PBS for 3 times. Donkey anti-Rabbit IgG H&L (Alexa Fluor® 555, 1:500 dilution) was then added and incubated for 2 hours and washed for 3 times with PBS. Next, Hoechst dye was added to stain cell nuclei. After 30 min, cells were rinsed with PBS three times and fluorescence density were determined in dark using a high-content analysis (HCA) instrument (PE Operetta), reflecting the change in receptor expression before and after drug intervention. Each well was examined under a fluorescence microscope with × 200 objective. Yellow and blue colors in stained areas in fields were recognized by detection system and calculated. The density was the positive area divided by the total area examined.

### Statistical analysis

Data from all the experiments were presented as mean ± SD. One-way ANOVA followed by Dunnett’s t-test were performed using GraphPad Prism 7 software (GraphPad Software, Inc., La Jolla, CA, USA). *p* < 0.05 is considered significant and *p* < 0.01 is considered highly significant.

## Results

### Meta-analysis of SXSM efficacy for bradycardia

**Fig. 1:**
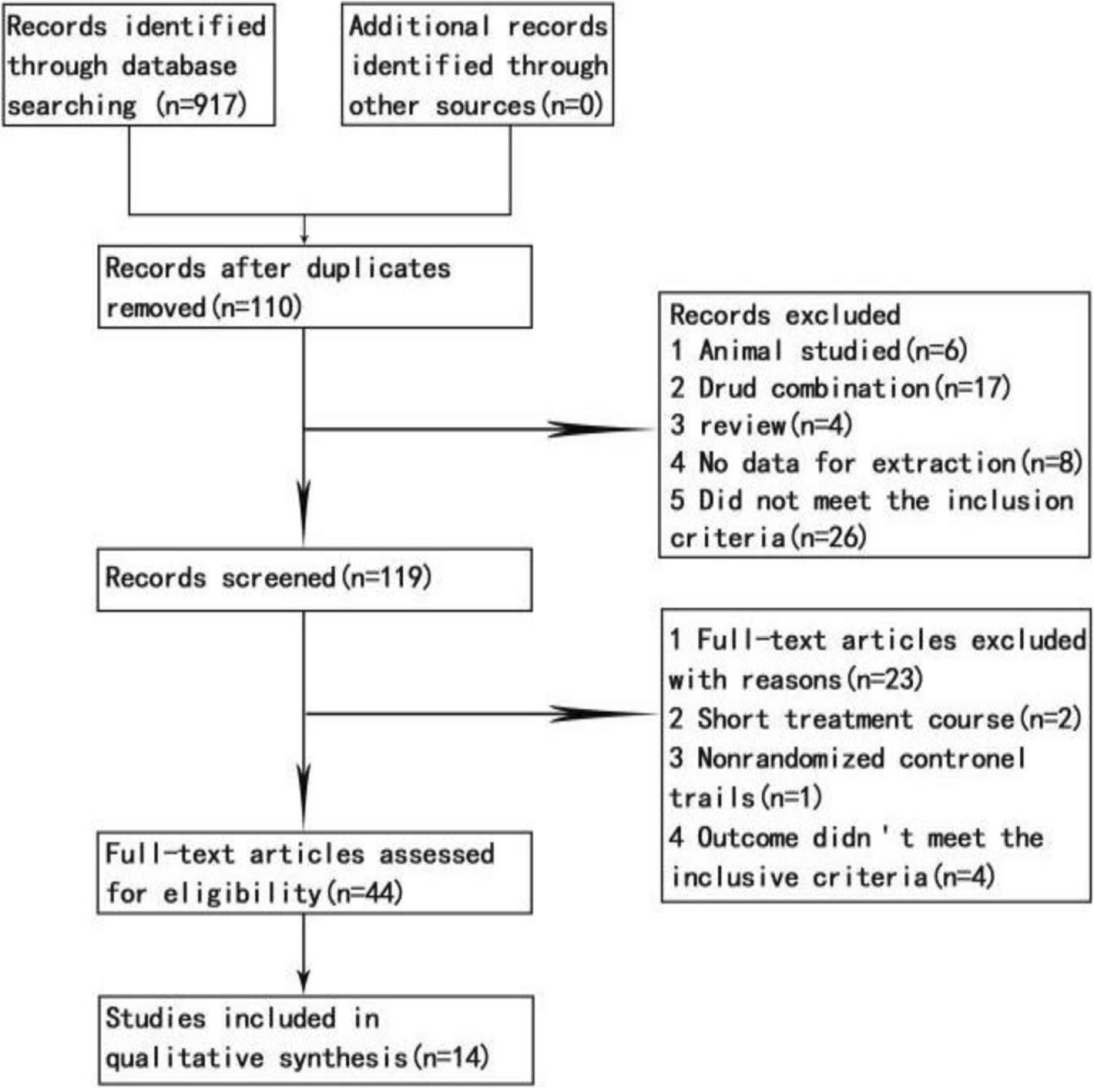
Flow chart of article selection process. By the criteria shown on the right, finally 14 articles were screened out

**Fig. 2:**
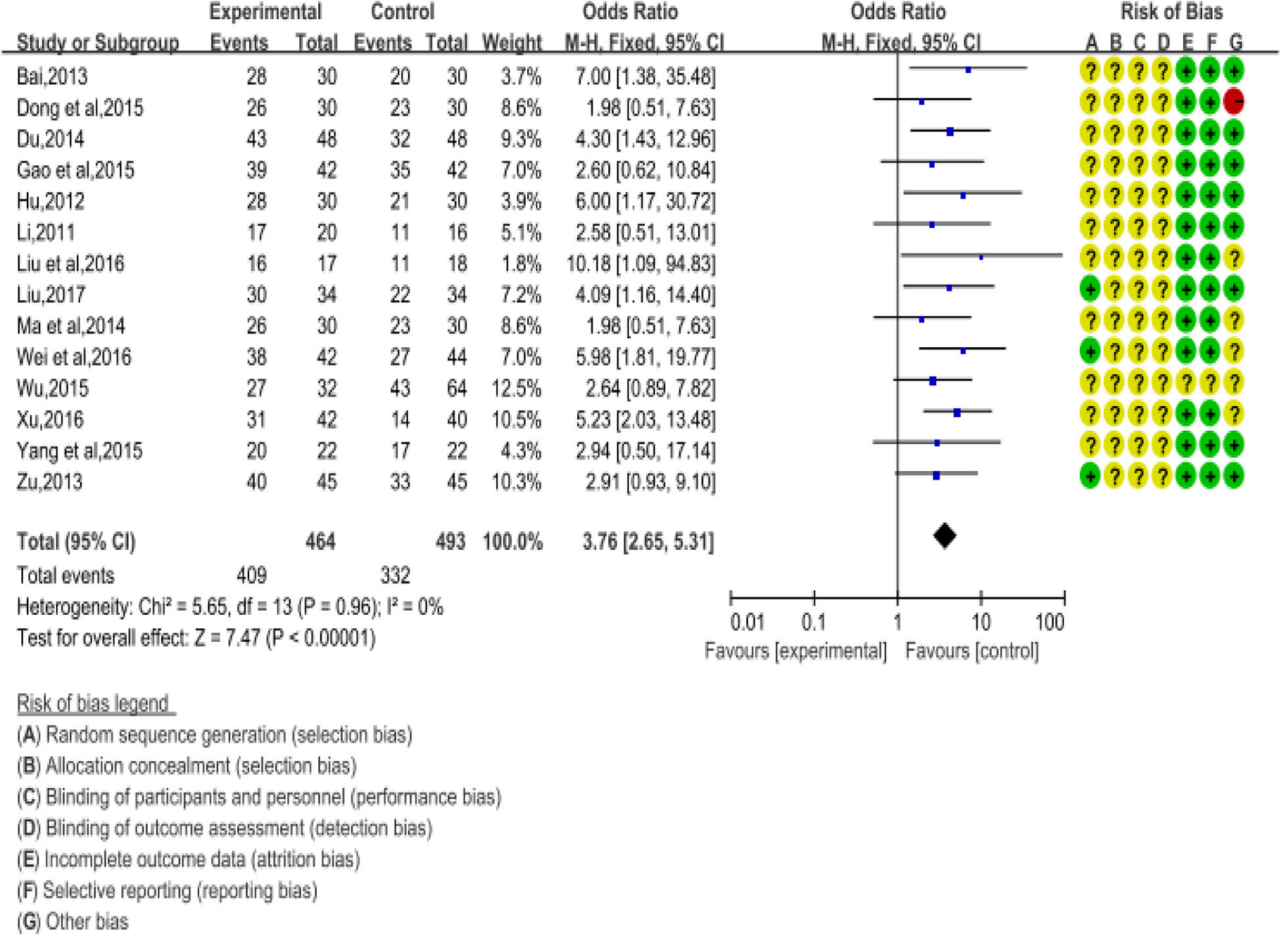
Assessment of the therapeutic effect of SXSM on patients with bradycardia. Analysis of heart rate shows that therapeutic effect percentage is 88.14% in SXSM group and 67.34% in the control group. Total 95%CI = 3.76. Heterogeneity I2 = 0%, Z = 7.47 (*P* < 0.00001) in total 957 cases. Risk of bias were evaluated for each research

**Fig. 3:**
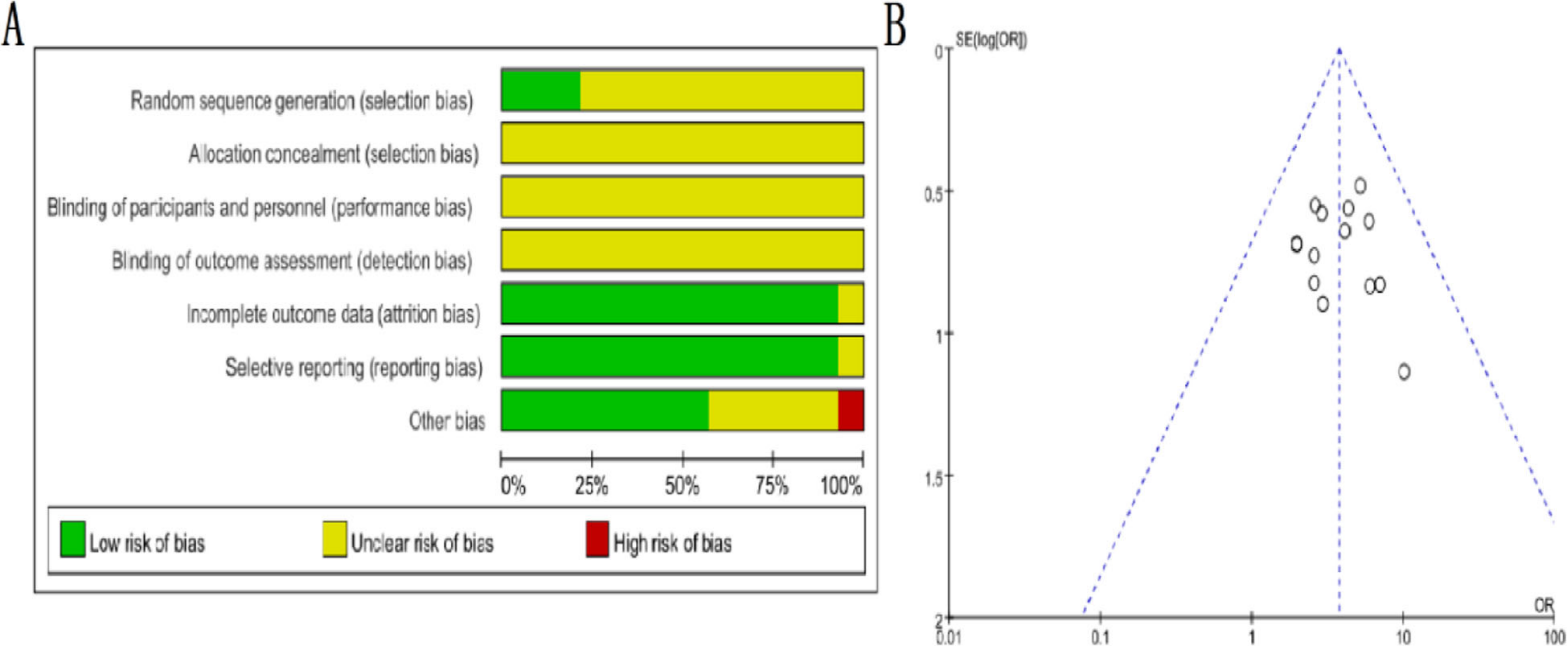
Risk of bias graph and funnel plot in meta-analysis. **a** Risk of bias graph. There were no high-risk literatures included in our study based on the seven assessment aspects of the Cochrane Collaboration Handbook standards. **b** Funnel plot. There was no small effect in the literature, indicating that the data obtained from different hospital subjects were basically the same, and the results were parallel comparable

A literature search using SXSM-related key words extracted a total of 917 records from various online databases. After a secondary screen by the above-mentioned criteria, 14 articles remain (Fig. 1 and Additional file 1: Table S1 and Table S2 for details). A total of 967 patients were included in the analysis. All these clinical studies used same administration methods (oral), for the same time course (4 weeks), and with same detection indexes of 24 h Holter dynamic electrocardiogram. In addition, plasma viscosity, pacemaker parameters, or complications were reported in some of the articles. Among the fourteen selected articles, 14 cases of side effects such as mildly dry mouth, nausea, and fatigue, were documented with none of the adverse events reported extremely serious. The risk factor assessment of the article was based on Cochrane’s seven assessment criteria, and the quality of the literature was examined on a random, double-blind basis. The results of each assessment are shown in Fig. 2 (More information available in supplemental materials). Overall, the Meta-analysis showed that the effective rate of the control group was 67.34%, while that of the SXSM treatment group was 88.14% by comparison, and total 95% CI = 3.76, I^2^ = 0, Z = 7.47 (*P* < 0.00001, Fig. 2). Thus, it indicated that SXSM has a better clinical curative outcome in bradycardia patients. A heterogeneity analysis by funnel plot showed that all the literatures included were concentrated in the middle of the funnel and proved that there was no small sample effect in this study (Fig. 3b).

### SXSM alleviated drug-induced bradycardia in an ex vivo cardiac model

To confirm the clinical findings, in vitro perfusion model of isolated rat hearts was used in which arrhythmias caused by neurotransmitter acetylcholine (Ach) were analyzed by heart rate (HR) recordings and electrocardiogram, and the effect of SXSM was assessed by drug administration at different time points. As shown in Fig. 3, compared with the control, Ach caused an apparent slow-down of the HR from 173 ± 52 to 67 ± 35. SXSM significantly accelerated the HR to the normal value (168 ± 61). On the other hand, LF\HF ratio was dramatically increased in Ach-treated heart compared with the controls (62 ± 15 vs 196 ± 37).

### Anti-bradycardia target prediction of SXSM components by network pharmacology analysis

**Fig. 4:**
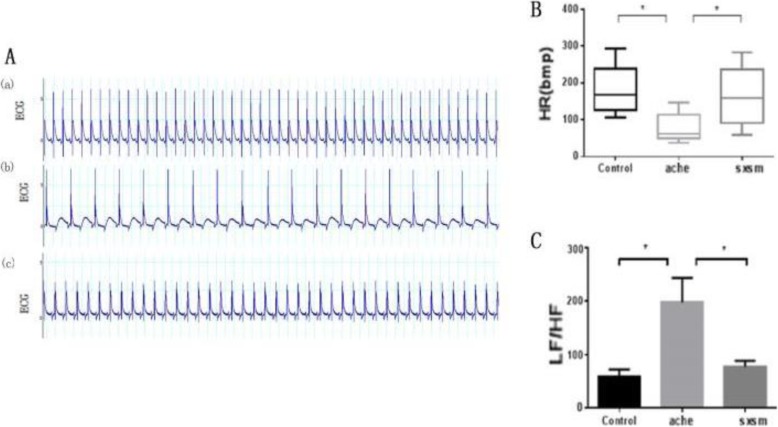
Effects of SXSM on acetylcholine-induced bradyarrhythmia in isolated rat heart. (**A**) representative ECG recordings of isolated and perfused rat hearts. Sample speed at 1k/s. Display of recorded results within 30 seconds. (a) untreated blank. (b)After acetylcholine perfusion. (c)After 1mg/ml SXSM perfusion. (**B**) Quantitation and statistical analysis of heart rate in each treatment group. There was no significant difference between the SXSM and blank groups. (**C**) Quantitation and statistical analysis of heart rate variability in each group. There was no significant difference between the SXSM and blank groups. Data are presented as mean ± SD (n =6). *P < 0.05

**Fig. 5:**
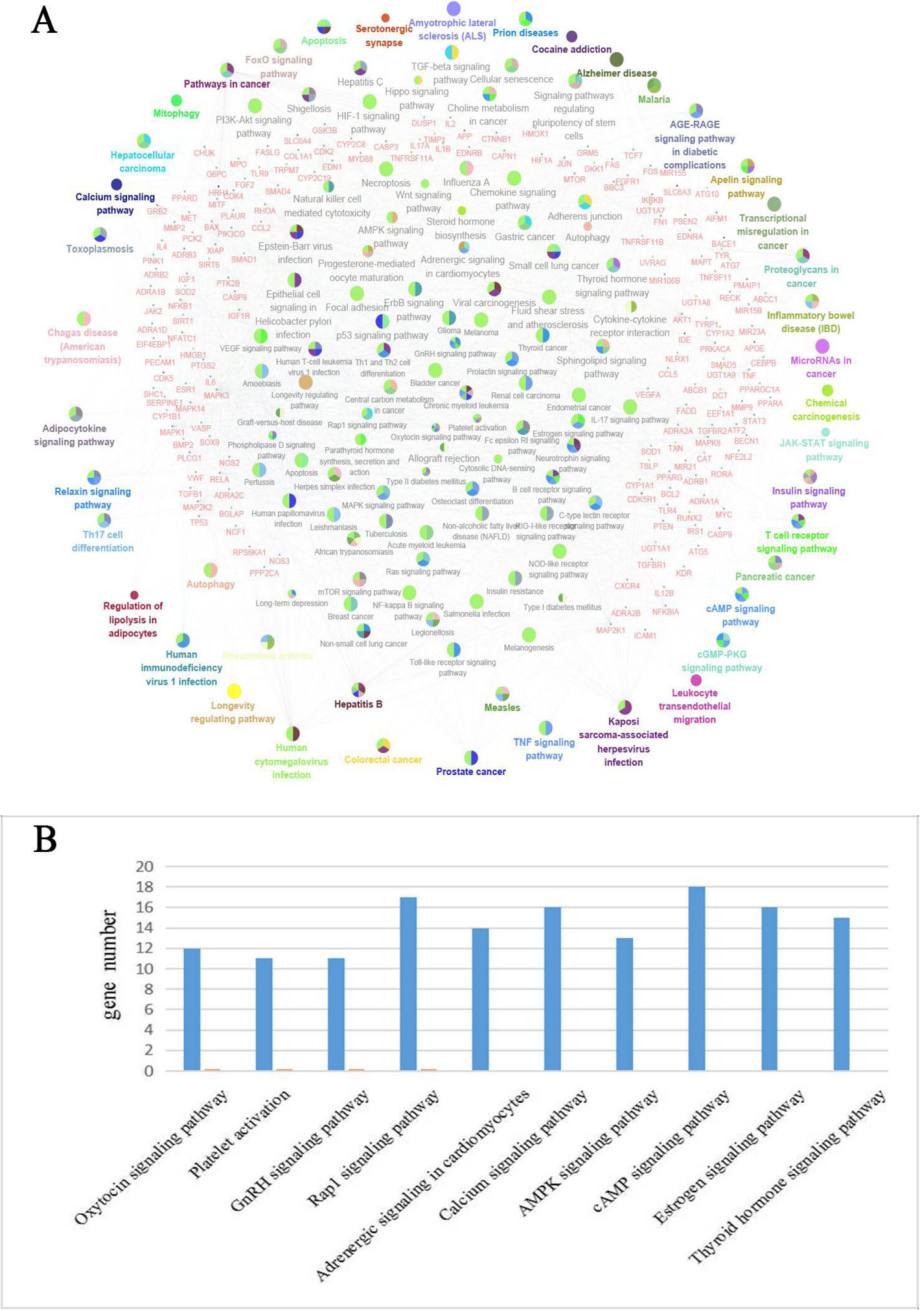
Classification and prediction of component-related targets. Function and pathway prediction were carried out based on the target library established with chemical components in SXSM. (**a**) KEGG analysis of compounds targets in SXSM. Small red dots represent targets, colored nodes represent enriched pathways and functions. All prediction are based on gene annotation of KEGG online databases. (**b**) Top10 predicted signaling pathway. The order of top pathways are ranked by −log(p-value) and number of targets included

Our previous work [13] identified 67 components in SXSM by UPLC-QTOF-MS/MS and UPLC-DAD. After mining literatures in PubMed and making supplement from online TCM databases, relevant targets were filtrated out to ensure each compound experimentally verified and database predicted with high-confidence. Please see Additional file 5 for protein interactions. Based on this targets database, we analyzed potential pathways affected by SXSM active components. PPI analysis and GO analysis in Cytoscape software were first used to show most probable drug-target interactions and related disease mechanisms. As shown in Fig. 4, gene targets (in red) and enriched pathways and functions (large colored dots) for arrhythmia were ranked according to the topological coefficients of network analysis and predicted *P* Value (−log). The top ten SXSM-targeted pathways associated with arrhythmia are in the order of: Oxytocin signaling, Platelet activation, GnRH signaling, Rap1 signaling, Adrenergic signaling in cardiomyocytes, Calcium signaling, AMPK signaling, cAMP signaling, Estrogen signaling, and Thyroid hormone signaling. As shown in Fig. 4 and Additional file 3: Table S3, possible interactions between SXSM components and bradycardia via specific pathways may include 14 SXSM components such as Epimedin B, Bavachalcone, and Ginsenoside Rb2 via Oxytocin signaling pathway; 17 components such as Ginsenoside Rb2, Salvianolic acid B, and Danshensu via platelet activation; 15 components such as Rosmarinic acid via GnRH signaling pathway; 12 components such as Ginsenoside Ro via Rap1 signaling pathway. Importantly, it was discovered that at least 19 SXSM components, such as Phenylpropanolamine, Pseudoephedrine, and Ephedrine, could play a role in regulating adrenergic signaling in cardiomyocytes. This prediction was further verified in Ingenuity Pathway Analysis (IPA) database by specifically analyzing bradycardia-related targets. G-protein coupled receptors, ion channels, and enzymes are among the major disease target types revealed. In all, 17 arrhythmia-related targets were identified, consistent with the expectation that a multi-target interaction mechanism may be involved in a multi-component medicine such as SXSM. In particular, the family of adrenergic receptors occupied a significant proportion of disease-related targets (Fig. 5) and was chosen for the validation test next.

### SXSM increased ADRB1 expression in cultured cardiomyocytes

**Fig. 6:**
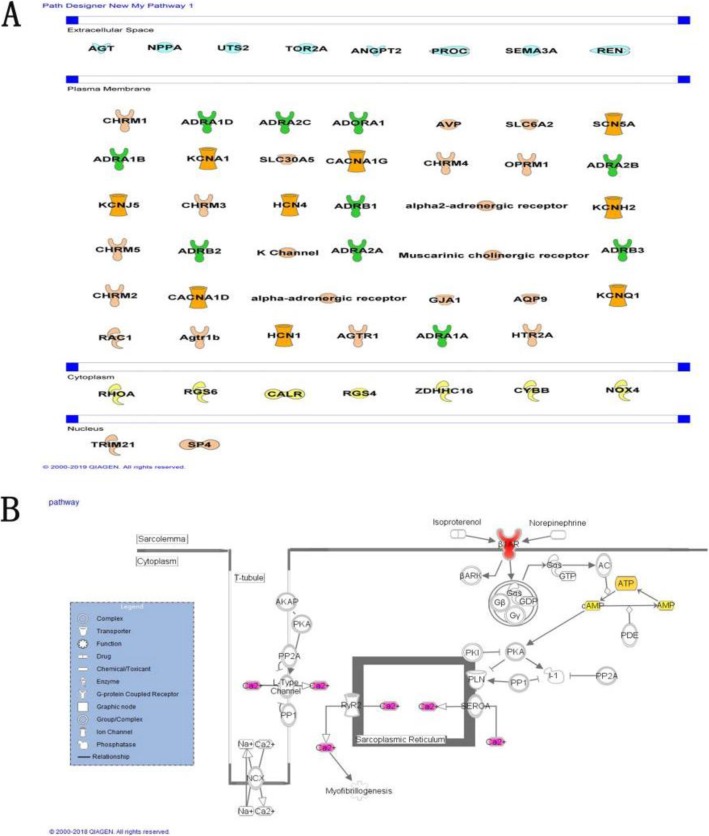
Target network of bradycardia. (**A**) Disease related genes are displayed by a classification of location in body. Differenttypes are represented by different shapes. Most of the targets exist in the cell membrane, mainly ion channels and G protein-coupled receptors. (**B**) In the overview of adrenergic signaling pathway in cardiomyocytes, the ADR family is at a relatively upstream position (colored in red). Other identified targets by SXSM components are colored in pink or yellow

**Fig. 7:**
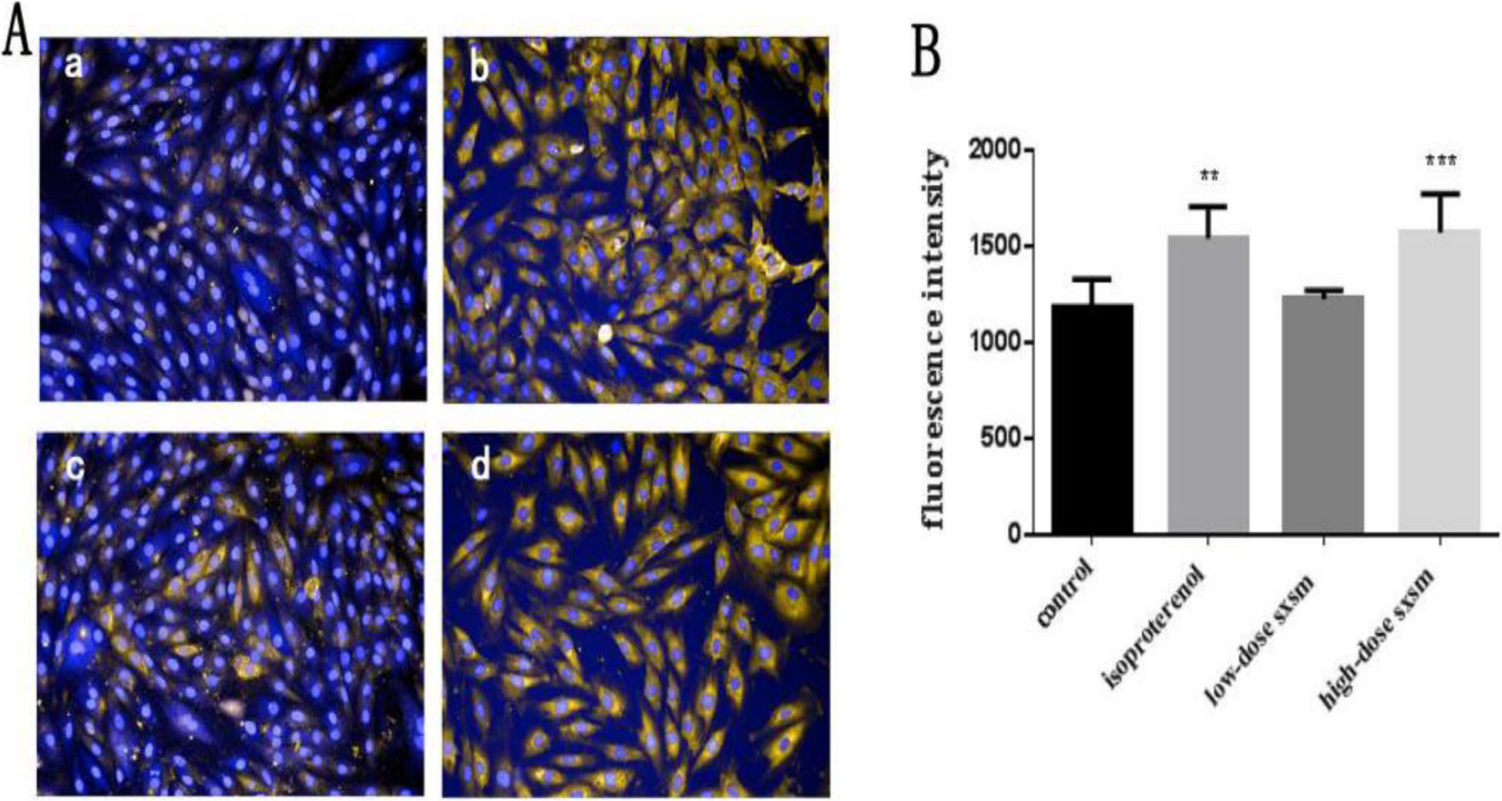
Effects of SXSM on ADRB1 expression in cultured cardiomyocytes. (**A**) representative images of H9c2 cells with different treatments. (a) untreated control, (b) positive control (isoproterenol, 10 uM), (c) low dose of SXSM (0.5 mg/ml), (d) high dose of SXSM (1 mg/ml). Blue fluorescence represents nucleus stained by Hoechst, and yellow fluorescence represents ADRB1 protein which exists both on cell membrane and in cytoplasm. The expression level of ADRB1 was up-regulated, indicating that SXSM regulated β1-adrenergic signaling pathway activation. (**B**) Bar graph quantitation of ADRB1 levels in H9c2 cells with different treatments. n≥5, **means *p*<0.001, ***means *p*<0.0001 compared with control

The effect of SXSM on adrenergic receptors was examined by immunofluorescence detection of ADRB1 expression in cultured H9c2 myocytes. As shown in Fig. 6, in the untreated control cells, the expression of ADRB1 was minimal whereas isoproterenol treatment increased ADRB1 level (*p* < 0.01). In comparison, high doses of SXSM prominently elevated the ADRB1 protein expression (*p* < 0.001) while low doses of SXSM had no effect (Fig. 7).

## Discussion

In this study, we first evaluated the clinical efficacy of SXSM for bradycardia with a meta-analysis of publicly available literature and confirmed it in an isolated rat heart model. Using network pharmacology analysis followed by experimental validation, we proved that mechanistically the anti-bradycardia effect of SXSM could be at least in part by upregulation of ADRB1 expression in cardiomyocytes. Innovations of this paper include: 1) an integrative study from clinical data meta-analysis to network pharmacology mechanism prediction and validation on a compound TCM formula; 2) discovery and confirmation that one of the possible anti-bradycardia mechanisms of SXSM is by regulating cardiac β1-adrenergic pathway.

In this study, we started from the clinical case analysis and found that SXSM indeed showed advantages in the treatment of bradycardia. It is worth noticing that while this research paper was in submission, a review article was published that also evaluated the effectiveness of some common TCM formulas, including SXSM, in treating patients with bradycardia [30]. The meta-analysis (including 1197 in SXSM group and 1133 in control group) was performed with a fixed- effect model as no significant heterogeneity was found (*I*^2^ < 50%, *P* > 0.1). It showed that SXSM was effective in treating bradycardia (RR: 1.33, 95% CI 1.27 to 1.39, *P* < 0.00001). Although the conclusions reached in this study and ours presented in this paper are consistent, our study design has eliminated the arrhythmia caused by other diseases during the screening process and chosen a fixed time course of 4 weeks. It is widely accepted that clinical and methodological source contribute to the heterogeneity of statistics. For our analysis, firstly, the dosage and time of SXSM oral liquid used by the patients were consistent. There were differences in the severity of bradycardia of patients in the fourteen included trials. Secondly, for most of these studies, the sample sizes were small and none of the trails were double blinded. Thirdly, doctor’s diagnose level and ECG equipment used in different hospitals could contribute additional variability. Therefore, high quality randomized clinical trials are needed to further clarify the SXSM efficacy as a complementary therapy for arrhythmia.

We used an isolated rat heart model of drug-induced bradycardia to experimentally confirm the SXSM effect. As expected, administration of acetylcholine increased LF\HF value, slowed heart rate, demonstrated an enhanced vagus nerve and sympathetic nerve activity in the model group. After treatment of SXSM, both the LF\HF ratio and heart rate reversed to normal level. We speculated that this phenomenon was because of the chemical composition in SXSM regulate the release of autologous acetylcholine by affecting the nerve activity, thereby maintaining its level in the steady state and finally increasing the heart rate (Fig. 3).

The complexity of the chemical composition in TCM formulas brings great challenges to pharmacological research. Network pharmacology of data mining and prediction has played a guiding role in the research of potential mechanism in recent years. It has become one of the most widely used techniques to explore compound TCM formulas. Our analysis showed that neuromodulation of SXSM is mainly via affecting the ADR receptor family (Fig. 4). Twenty-nine of these targets were common for both disease and drug chemical database, which were listed in Additional file 4: Table S4. Compounds originated from Herba Ephedrae Sinicae (mahuang) and Herba Epimedii Brevicornus (yinyanghuo) contribute mostly to the influence of ADR receptor family, particularly ADRB1 protein, which is known to affect heart rhythm by neuronal regulation. The rest compounds from other six herbs tend to play a role in humoral regulation that is associated with cytokines in the process of inflammation and injury, such as TNF, TGFβ1, IL6, and SIRT6. This indicated that SXSM could be considered to treat those patients who suffered from cardiac hypertrophy, heart failure, myocardium inflammation and thrombus in order to prevent complication of bradycardia.

Holistic approach is the core principle of Traditional Chinese medicine, which is also supported by our findings in this study. For example, the effect of SXSM on elevating heart rate in bradycardia not only include heart-specific actions such as β1-Adrenergic receptor activation1, but also multiple neuronal regulation in brain and systemic inflammatory response. However, a holistic action of a compound formula is composed of multiple compound-specific actions – only when we understand these individual compound-specific action and integrate them into a coordinated network, that we will be fully interpret a holistic action. Our pathway prediction suggested a potential mechanism of multi-target regulation of arrhythmia by a TCM formula. The top10 predicted signaling pathways revealed significant functional implications. For example, oxytocin (OT) is a common neurotransmitter released by paraventricular nucleus (PVN). It is related to regulation of pain perception. The heart protective effects of OT are mediated through opening the mitoKATP channels [31]. Thrombosis is common in cardiovascular and cerebrovascular diseases and arrhythmia is tightly associated with myocardial ischemia and myocardial infarction. Changes of platelet reactivity to thrombin observed after restoration of sinus rhythm in patients proved that arrhythmia intrinsically leads to increased reactivity of platelets [32]. Gonadotropin-releasing hormone (GnRH) is a neurotransmitter that reported to have connection with inflammation signaling in cardiomyocytes [33]. Epac2-Rap1 signaling regulates reactive oxygen species production and susceptibility to arrhythmia [34]. Analysis of Rap1 signaling pathway indicates that SXSM may affect cellular interaction and attenuate mitochondrial ROS production and reduce arrhythmia susceptibility. β-adrenergic regulation of late Na^+^ current during cardiac action potential and its regulation of arrhythmia was recently reported [35]. Actually, calcium signaling and cAMP signaling pathways are partially overlapping and closely related to adrenergic signaling pathway. Abnormal calcium handling results in a predisposition to atrial fibrillation [36]. Our prediction of AMP-activated protein kinase (AMPK) signaling pathway suggests that SXSM may also play a role in affecting energy metabolism in cardiomyocytes. AMPK activation has attracted more and more attention in the development of new therapeutic strategies for cardiometabolic disease [37]. In summary, we believe that from the SXSM components identified so far, its anti-bradycardia effect are mainly focused on regulating the release of neurotransmitters, causing nervous system excitement. Supplemented by promoting blood flow and increasing energy metabolism, finally increased the heart rate.

The new guideline on the evaluation and management of patients with bradycardia [38] demonstrated that for patients with acute bradycardia of AV block, who are at low likelihood of coronary ischemia but associated with symptoms or hemodynamic compromise, β-agonist drugs (such as isoproterenol, dopamine, dobutamine, or epinephrine) are recommended treatment to increase heart rate and improve symptoms. Cardiac β-adrenergic pathway is one of the classic pathways for regulating arrhythmias. β1-Adrenergic receptor (β1-ARs) activation can provoke arrhythmia mediated by activate cAMP-dependent Ca2^+^ release from the sarcoplasmic reticulum (SR) via phosphorylation of RyR2. In the updated classification of antiarrhythmic drugs published in 2018, β-receptor modulators are classed as type II antiantiarrhythmic drugs [39]. What’s more, cAMP can activate both protein kinase A (PKA) and the exchange protein directly triggered by cAMP (Epac) which mediates β1-AR-induced arrhythmia via CaMKII or RyR2 phosphorylation, and activates LTCC [26]. Some prior studies indicated that SXSM could regulate RYR2\PLB protein [10], which may cause LTCC activation via L-calcium channel. β-adrenergic pathway has been a frequent focus for research of cardiac diseases like heart failure and hypertrophy [40, 41]. Our network pharmacology prediction and experimental validation confirm that SXSM may play a therapeutic role through this classic pathway, providing guidance for its rational clinical application.

It is worth noticing that over the recent years, adrenaline has been gradually recognized to also inhibit inward potassium current I_ks_, activate acetylcholine coupled inward potassium current I_ache_, regulate multiple ion channels, and coordinate antiarrhythmic drugs. Ion channels on cellular membranes play a direct and crucial role in the formation of normal heart rhythm, whose open and close process leads to the generation of action potential (AP). Once the dynamic equilibrium on ion channels is broken, arrhythmia may occur. Accordingly, most of the current anti-arrhythmic drugs are ion channel modulators. Electrophysiological research on compounds present in the eight Chinese herbs in SXSM has not been carried out extensively so far, which limited the power of our network pharmacology analysis. However, it also justifies that further investigation of SXSM should include electrophysiological exploration of its components on ion channel target.

In summary, although traditional Chinese herbal medicine has a long history of the treatment for cardiac rhythm symptoms dating back to as early as in the Han dynasty of China, it has not received enough attention due to the lack of in-depth clinical to basic science investigations. Our future work will identify active chemical components and reveal their specific mechanisms of SXSM, hoping to provide alternative therapeutic options to arrhythmia patients who could not accept pacemaker implantation.

## Conclusions

In this study, we established the efficacy of Shenxian-Shengmai Oral Liquid for bradycardia by both meta-analysis of clinical data and ex vivo study in rat heart. We identified a number of critical pathways including the critical β1-adrenergic signaling pathway and mechanisms that could be affected by SXSM. Our approaches integrated clinical, experimental and network pharmacology analyses to reveal the compounds-targets-pathways-disease connections and uncover the underlying mechanisms of a compound herbal medicine, which could be applied to other multi-component traditional Chinese medicine investigation where ample clinical data are accumulated but advanced mechanistic studies are lacking.

## Supplementary information

**Additional file 1: Table S1.** Quality assessment of the included randomized controlled trials on the seven assessment aspects of the Cochrane Collaboration Handbook standards.

**Additional file 2: Table S2.** Characteristics of meta-analysis studies, including patient groups; diagnosis criteria of bradycardia; mode of administration and course of treatment; selection of drugs in the control group; evaluation indicators and adverse effects.

**Additional file 3: Table S3.** Pathway-Gene-Compounds relationship of SXSM. Top ten pathways and *P* values predicted by network pharmacology, gene targets involved in each pathway, and compounds that could interact with the targets theoretically.

**Additional file 4: Table S4.** Common targets for disease and drug components.

**Additional file 5: Figure S1.** Relationship between disease-related proteins.

## Data Availability

The datasets used and/or analyzed in the current study are available from the corresponding author on request.

## Abbreviations

ADRB1: Beta-1 adrenergic receptor
bpm: Bite per minute
CIED: Cardiac implantable electronic device
CVDs: Cardiovascular diseases
ECG: Electrocardiogram
HR: Heart rate
MESH: Medical Subject Headings
SXSM: Shenxian-Shengmai oral liquid
TCM: Traditional Chinese medicine
