# Supplementary figures and images for: Clinical evidence-guided network pharmacology analysis reveals a critical contribution of β1-adrenoreceptor upregulation to bradycardia alleviation by Shenxian-Shengmai
Source: BMC Complement Altern Med. 2019 Dec 10;19:357. doi: 10.1186/s12906-019-2769-0 (PMC6902583; doi:10.1186/s12906-019-2769-0)

**Figure S1**


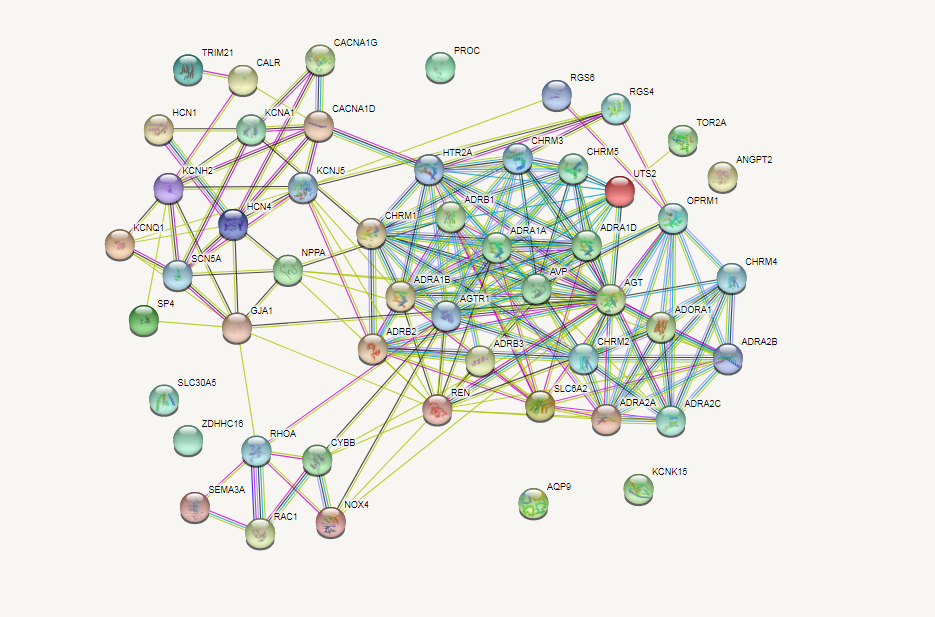

Supplement: Supplementary file 5 — Additional file 5: Figure S1. Relationship between disease-related proteins. [file 12906_2019_2769_MOESM5_ESM.docx]
